# Supplementary material for: In vivo imaging of the human brain with the Iseult 11.7-T MRI scanner
Source: Nat Methods. 2024 Oct 17;21(11):2013–6. doi: 10.1038/s41592-024-02472-7 (PMC11541209; doi:10.1038/s41592-024-02472-7)
Supplement: Supplementary file 1 — Reporting Summary [file 41592_2024_2472_MOESM1_ESM.pdf]

nature portfolio

Corresponding author(s): Denis Le Bihan

Last updated by author(s): Jul 25, 2024

# Reporting Summary

Nature Portfolio wishes to improve the reproducibility of the work that we publish. This form provides structure for consistency and transparency in reporting. For further information on Nature Portfolio policies, see our Editorial Policies and the Editorial Policy Checklist.

## Statistics

For all statistical analyses, confirm that the following items are present in the figure legend, table legend, main text, or Methods section.

| n/a | Confirmed | |
|---|---|---|
| ☐ | ☒ | The exact sample size (*n*) for each experimental group/condition, given as a discrete number and unit of measurement |
| ☐ | ☒ | A statement on whether measurements were taken from distinct samples or whether the same sample was measured repeatedly |
| ☐ | ☒ | The statistical test(s) used AND whether they are one- or two-sided<br>*Only common tests should be described solely by name; describe more complex techniques in the Methods section.* |
| ☒ | ☐ | A description of all covariates tested |
| ☒ | ☐ | A description of any assumptions or corrections, such as tests of normality and adjustment for multiple comparisons |
| ☐ | ☒ | A full description of the statistical parameters including central tendency (e.g. means) or other basic estimates (e.g. regression coefficient) AND variation (e.g. standard deviation) or associated estimates of uncertainty (e.g. confidence intervals) |
| ☐ | ☒ | For null hypothesis testing, the test statistic (e.g. $F$, $t$, $r$) with confidence intervals, effect sizes, degrees of freedom and $P$ value noted<br>*Give P values as exact values whenever suitable.* |
| ☒ | ☐ | For Bayesian analysis, information on the choice of priors and Markov chain Monte Carlo settings |
| ☒ | ☐ | For hierarchical and complex designs, identification of the appropriate level for tests and full reporting of outcomes |
| ☐ | ☒ | Estimates of effect sizes (e.g. Cohen's *d*, Pearson's *r*), indicating how they were calculated |

*Our web collection on statistics for biologists contains articles on many of the points above.*

## Software and code

Policy information about availability of computer code

| | |
|---|---|
| Data collection | the Attentional Network Task experiment was ran with a custom python code available at https://github.com/chrplr/Posner_attention_networks_task (note: "GRAPE pulse" in not a code, but the name of a standard MRI sequence) |
| Data analysis | behavioral data were analysed with custom code in the R language available at http://github.com/chrplr/prems |

For manuscripts utilizing custom algorithms or software that are central to the research but not yet described in published literature, software must be made available to editors and reviewers. We strongly encourage code deposition in a community repository (e.g. GitHub). See the Nature Portfolio guidelines for submitting code & software for further information.

## Data

Policy information about availability of data

All manuscripts must include a data availability statement. This statement should provide the following information, where applicable:

- Accession codes, unique identifiers, or web links for publicly available datasets
- A description of any restrictions on data availability
- For clinical datasets or third party data, please ensure that the statement adheres to our policy

All behavioral data supporting the findings of this study are available at http://github.com/chrplr/prems

## Human research participants

Policy information about studies involving human research participants and Sex and Gender in Research.

| | |
|---|---|
| Reporting on sex and gender | No sex or gender analysis was performed. |
| Population characteristics | Group 0T : n=20 24.2 ± 4.8 year 8 males<br>Group 11.7T : n=20 23.6 ± 6.0 year 7 males |
| Recruitment | Subjects were recruited freely through local annoucements. No bias has to be reported. |
| Ethics oversight | This study was approved by the French “Agence Nationale de Sûreté et du Médicament” (ANSM) regulatory body and a national ethics committe (IDRCB 2022-A02321-42) as stated in the manuscript. |

Note that full information on the approval of the study protocol must also be provided in the manuscript.

# Field-specific reporting

Please select the one below that is the best fit for your research. If you are not sure, read the appropriate sections before making your selection.

☒ Life sciences ☐ Behavioural & social sciences ☐ Ecological, evolutionary & environmental sciences

For a reference copy of the document with all sections, see nature.com/documents/nr-reporting-summary-flat.pdf

# Life sciences study design

All studies must disclose on these points even when the disclosure is negative.

| | |
|---|---|
| Sample size | The sample size was determined by the regulation agency approval to test MRI at 11.7T (world premiere). 20 subjects were exposed to 11.7T and 20 subjects to 0T. Subjects were not aware of the presence or not of the magnetic field. This is stated in the manuscript. |
| Data exclusions | All included subjects are analyzed. Exclusion criteria (as stated in the Methods section of the manuscript) were: usual counter-indications for MRI (pacemakers, implants, metallic objects, tattoos, claustrophobia etc), age (below 18 and above 40yo), anxiety (Hamilton) score >20 |
| Replication | N/A. This study allowed only 20 subjects to be scanned, as mentioned in the manuscript. |
| Randomization | N/A Data acquisitions were performed in the order subjects came to be enrolled as volunteers for this study. The first 20 subjects were "scanned" at 0T, when the magnet was off (during several weeks). The next 20 subjects were scanned at 11.7T when the magnet was on. Subjects were not aware of the field value (0 or 11.7T), as stated in the manuscript. |
| Blinding | N/A. For the above reason blinding was not possible for the data acquisition. |

# Reporting for specific materials, systems and methods

We require information from authors about some types of materials, experimental systems and methods used in many studies. Here, indicate whether each material, system or method listed is relevant to your study. If you are not sure if a list item applies to your research, read the appropriate section before selecting a response.

## Materials & experimental systems

| n/a | Involved in the study | |
|---|---|---|
| ☒ | ☐ | Antibodies |
| ☒ | ☐ | Eukaryotic cell lines |
| ☒ | ☐ | Palaeontology and archaeology |
| ☒ | ☐ | Animals and other organisms |
| ☒ | ☐ | Clinical data |
| ☒ | ☐ | Dual use research of concern |

## Methods

| n/a | Involved in the study | |
|---|---|---|
| ☒ | ☐ | ChIP-seq |
| ☒ | ☐ | Flow cytometry |
| ☐ | ☒ | MRI-based neuroimaging |

# Magnetic resonance imaging

## Experimental design

| | |
|---|---|
| Design type | Structural MRI |
| Design specifications | N/A (not a fMRI study). |
| Behavioral performance measures | N/A (only structural MRI was performed, as stated in the manuscript). |

## Acquisition

| | |
|---|---|
| Imaging type(s) | Structural |
| Field strength | 11.7T |
| Sequence & imaging parameters | All details are provided in the Methods section |
| Area of acquisition | Head |

Diffusion MRI ☐ Used ☒ Not used

## Preprocessing

| | |
|---|---|
| Preprocessing software | N/A. It is a structural MRI study |
| Normalization | *If data were normalized/standardized, describe the approach(es): specify linear or non-linear and define image types used for transformation OR indicate that data were not normalized and explain rationale for lack of normalization.* |
| Normalization template | *Describe the template used for normalization/transformation, specifying subject space or group standardized space (e.g. original Talairach, MNI305, ICBM152) OR indicate that the data were not normalized.* |
| Noise and artifact removal | *Describe your procedure(s) for artifact and structured noise removal, specifying motion parameters, tissue signals and physiological signals (heart rate, respiration).* |
| Volume censoring | *Define your software and/or method and criteria for volume censoring, and state the extent of such censoring.* |

## Statistical modeling & inference

| | |
|---|---|
| Model type and settings | N/A. It is a structural MRI study |
| Effect(s) tested | *Define precise effect in terms of the task or stimulus conditions instead of psychological concepts and indicate whether ANOVA or factorial designs were used.* |

Specify type of analysis: ☐ Whole brain ☐ ROI-based ☐ Both

| | |
|---|---|
| Statistic type for inference (See Eklund et al. 2016) | *Specify voxel-wise or cluster-wise and report all relevant parameters for cluster-wise methods.* |
| Correction | *Describe the type of correction and how it is obtained for multiple comparisons (e.g. FWE, FDR, permutation or Monte Carlo).* |

## Models & analysis

| n/a | Involved in the study |
|---|---|
| ☒ | ☐ Functional and/or effective connectivity |
| ☒ | ☐ Graph analysis |
| ☒ | ☐ Multivariate modeling or predictive analysis |
